# Arsenical Treatments in Compound Cavities Confined with Cotton and Sandarac Varnish

**Published:** 1904-11

**Authors:** C. M. Baldwin

**Affiliations:** Chicago.


### ARSENICAL TREATMENTS IN COMPOUND CAVITIES CONFINED WITH COTTON AND SANDARAC VARNISH.

By C. M. Baldwin, D. D. S., Chicago.

(Written for the American Journal of Dental Science.)

In compound approximal cavities in bicuspids and molars extending to or below the gums, when for any reason anesthesia methods are contra-indicated for the removal of live pulp tissue, arsenic will continue to be used.

Probably all dental students are instructed to carefully cover arsenical treatments with either cement or guttapercha, and cautioned to never use cotton for such a purpose.

In this class of cavities when the gums cover more or less of the cavity, it requires considerable time and care to confine arsenic with cement or guttapercha, and too often arsenical poisoning of the gums results after every effort has been made to prevent it.

Many times one or more sittings will be required to pack the cavity with cotton or guttapercha to force the gums out of the cavity, or the gums must be cut away, when it will be more difficult to place and confine the arsenic at that sitting without leakage resulting.

Often it is exceedingly difficult, many times it is impossible to adjust the rubber dam in these cases until some preliminary measure has been taken to exclude the gums from the cavity, or it may be necessary to fill a portion of the cavity to temporarily or permanently raise the gingival margin, so that the saliva may be excluded by the rubber.

Can cotton and sandarac varnish be used successfully in this class of cavities to control arsenical preparations from one to three days?

Several years ago at a charity clinic Dr. C. T. Gramm used cotton and sandarac varnish to cover an arsenical treatment.

The writer has gradually come to use this simple method for the same purpose in all large cavities as mentioned above, to which may be added occasional cavities in the six anterior teeth where the same principles are involved, and as yet without a single leakage having been found.

*Cotton and Sandarac Method.*—Excavate and medicate the cavity as desired, but do not cut margins too much in large open cavities, as they help retain the plug.

The rubber is never applied in these cases, but dryness is secured by the use of cotton rolls, the Hare mouth prop and the saliva ejector, as may be needed.

The cavity and surrounding parts should be thoroughly dried, including the surface of the tooth facing the cavity and the gums between, and should be kept so until the packing is completed, the varnish applied and congealed.

When necessary, the gums between the teeth may be treated with alcohol, chloroform, 4 per cent. trichlor acetic acid or adrenalin chloride 1-1000 to control moisture.

Cotton pellets of suitable size should be prepared, so that from two to four will be sufficient to pack the cavity and space to the adjoining tooth.

If a moistened arsenical preparation is used, do not put the pellet of cotton into it, but with a small pointed instrument place the arsenic on a very small dry piece of cotton, so that the pellet will not be completely moistened if pressure is applied.

In most cases an adjoining tooth aids the packing and retention of the plug, and all such cases should be partially packed before the arsenic pellet is placed.

Pack one or two large pellets of dry cotton between the adjoining tooth and the side margins of the cavity, so that the cotton is wedged in firmly, compressing the gums, filling the space to the contact point, and extends on the floor and walls of the cavity to the exposed pulp.

The arsenic pellet is now easily placed, being supported by the packed cotton, and one or two more pieces of cotton are packed, bridging over the pulp to the walls of the cavity, completing the plug, when the varnish should be applied immediately at the gum line, afterwards on the occlusal surface, but stop short of saturation, as the inner portion of the plug nearest the pulp and arsenic pellet should remain unvarnished. The last step is to lightly compress the varnished surface of the plug toward the margins with wet cotton.

When there is no adjoining tooth, the same routine is followed, except that the wedging in packing is within the cavity, and the arsenic pellet may sometimes be placed before the packing is begun. All packing should be directed away from the exposed pulp, against the adjoining tooth, gums and walls of the cavity, which with the bridging effect of the last one or two pellets makes a large dome-like plug more or less hollow or unpacked in the region of the pulp, so that there will not be any pressure upon it. When the pulp is not exposed, the plug should be solid throughout.

See that the plug is not touched by the occluding teeth, and caution the patient as carefully as the combination of patient and cavity may require.

In this class of cavities, upon removal of the arsenical treatment, wash the cavity and gums thoroughly, and apply dialysed iron to the gums nearest the cavity, regardless of the form of stopping that may have been used.

If the gums cover the gingival margin but slightly, pack the cavity as directed above, placing the arsenic pellet on the side of the exposed pulp farthest from the gums.

When the pulp is not exposed, cover the entire floor of the cavity with well packed cotton, before placing the arsenic near the floor at the axial wall or at some higher position on the axial wall.

When considerable gum tissue occupies the floor of the cavity the packing will vary somewhat according to the position of the exposed pulp, whether to pack ignoring the gums if the exposure be high on the axial wall, or whether it will be necessary to raise the gumflap, packing so as to force the tissue out of the cavity, but in every case there must always be a substantial well packed layer of dry cotton between the gums and the arsenic pellet.

In cases where the gums are more or less in the cavity, use 4 per cent. tri chlor acetic acid, or adrenalin chloride 1-1000 to constrict the gum tissue, so that the cotton will not be perceptibly moistened when packed on it.

Since using this method the lance is very seldom used to remove hypertrophied gum tissue from cavities, and no case can be recalled where the arsenic was not applied at the first packing of the cavity, except when previous medication was necessary to relieve pain.

Dry cotton is sufficient to plug test tubes in pathological laboratories, and the success of the cotton sandarac method is due to the fact that no moisture is introduced into the cavity except on the arsenic pellet and that the varnished plug excludes the saliva, so that there is no vehicle to carry the arsenic to the gums, as dry cotton seems to be a perfect non conductor.

The instrumentation necessary to apply cement or guttapercha to the gingival margins of these cavities and avoid irritating the pulp, has undoubtedly caused sufficient moisture in many cases to account for the arsenical poisonings often attributed to the shrinkage or porosity of the stopping.

*Summary.*—The simplicity of the cotton sandarac method permits one to quickly and carefully apply it, thus reducing to the minimum any chances of the saliva entering the cavity while the treatment is being inserted.

Placing and retaining the arsenic in position is more certainly and easily accomplished.

There is very much less chance of irritating the pulp by pressure or from contact of stopping, than if cement or guttapercha is used.

As devitalizing the pulp is but a means to the desired end of permitting its removal, the cotton-sandarac method should appeal to one, because it is a great economizer of time and energy for both patient and operator, and permits the same end to be attained.

